# Ostracism, cortisol reactivity, and motivation for high-calorie food in children and adolescents with obesity

**DOI:** 10.1038/s41366-025-01824-3

**Published:** 2025-09-24

**Authors:** Anna Felnhofer, Andreas Goreis, Lisa Weiss, Helmuth Haslacher, Charlotte Nigmann, Gabriele Skacel, Rodrig Marculescu, Paul L. Plener, Susanne Greber-Platzer, Oswald D. Kothgassner

**Affiliations:** 1 Department of Pediatrics and Adolescent Medicine, Division of Pediatric Pulmonology, Allergology and Endocrinology, Comprehensive Center for Pediatrics, Medical University of Vienna, Vienna, Austria; 2 Department of Child and Adolescent Psychiatry, Comprehensive Center for Pediatrics, Medical University of Vienna, Vienna, Austria; 3 Department of Laboratory Medicine, Medical University of Vienna, Vienna, Austria; 4 Department of Child and Adolescent Psychiatry and Psychotherapy, University of Ulm, Ulm, Germany

**Keywords:** Obesity, Risk factors, Obesity

## Abstract

**Objective:**

Experiencing ostracism (i.e., social exclusion) may impact self-regulatory eating behaviors, particularly in youths with excess weight. Yet, research in pediatric patients with obesity is lacking. Hence, we examined the effect of Virtual Reality(VR)-based ostracism on motivation for food in children and adolescents with BMI ≥97^th^ percentile.

**Methods:**

In a randomized experimental between-subject design, forty-one patients (M_age_ = 13.37 years, 46% female) with a diagnosis of obesity (ICD-10: E66) were randomized to a social exclusion or inclusion condition in a VR-Cyberball-paradigm. Patients’ salivary cortisol, heart rate and heart rate variability were assessed. Furthermore, we measured patients’ motivation to consume high-calorie food, their prosocial behavior, their self-reported urge to eat and subjective stress.

**Results:**

Results indicate that the experience of social exclusion in youths with obesity leads to a blunted salivary cortisol response; in contrast, no effects of social exclusion on the sympathetic nervous system were observed. Social exclusion was associated with an increased perceived threat to fundamental social needs. Similarly, ostracized participants demonstrated heightened self-regulatory behaviors regarding their motivation for high-calorie food intake, selecting fewer grams of sweets following social exclusion. Furthermore, ostracism tended to increase helping behavior post-exclusion, although this effect was not significant. Self-reported urge to eat and stress levels during the experiment showed no significant effect.

**Conclusion:**

Ostracism-induced reduction of motivation for food suggests that affiliative behaviors like increasing compliance regarding eating behaviors may play a role in youths with obesity with BMI ≥97^th^ percentile in the context of social stress. Future research should explore the broader social context, including family and friends, to better understand the dynamics between social stress, physiological reactivity, and self-regulatory behaviors in treating obesity.

**Clinical trial registration:**

As this study does not constitute a clinical trial, the study design and analyses plans were not preregistered.

## Introduction

Childhood obesity is defined by the WHO as excessive fat accumulation and is classified as a BMI (kg/m²) ≥97^th^ percentile for age and sex, and as severe obesity when the BMI is ≥99^th^ percentile [1, 2]. One factor in the development and maintenance of childhood obesity is eating behavior, particularly the tendency to over-consume high-calorie foods [3, 4]. Research suggests that individuals with obesity are more likely to use eating as a coping mechanism for stress compared to those with an average weight [5]. Among stressors, the experience of ostracism – characterized by social exclusion, rejection, or being ignored – is considered highly impactful for all social beings [6]. Particularly social exclusion has been shown to – on the one hand – undermine fundamental social needs such as belonging and control [7, 8] and – on the other hand – to deplete attentional resources essential for self-regulation. Consequently, it has been suggested that social exclusion may also influence food intake [9]. Even brief ostracism episodes have been found to increase the likelihood of consuming unhealthy foods in average-weight adults [10, 11]. Compared to adults, adolescents may be even more vulnerable to ostracism-triggered comfort eating due to their heightened rejection sensitivity [12]. This vulnerability may be particularly pronounced among individuals with obesity, who not only face social exclusion but also experience weight-related stigmatization in their personal relationships [13].

Initial studies in adolescents show that experimental ostracism indeed increases motivation for food and food intake [5, 9, 14]. Yet, these past samples were heterogeneous and included no children and/or adolescents with more severe forms of obesity (i.e., with BMI ≥99^th^ percentile). This is notable, as research suggests that children with severe obesity may experience higher levels of emotional eating and stress [15], as well as lower overall health-related quality of life than those with less severe obesity [16]. Additionally, no study has so far considered physiological reactivity in ostracized children and adolescents with obesity, although there is strong evidence for the impact of social exclusion on the hypothalamic–pituitary–adrenal (HPA) axis and the autonomic nervous system (ANS) [17]. For instance, the use of another type of stressor, a social-evaluative paradigm (TSST [18]), has resulted in elevated cortisol levels, which in turn increased food intake in adults [19] and children [20] with obesity.

Given the lack of research, particularly in children and adolescents with more severe forms of obesity, we aimed to explore how a brief social exclusion episode impacts self-regulation with regards to high-calorie foods in children with BMI ≥97^th^ percentile. To replicate realistic social interactions, we used virtual reality (VR), known for its high ecological validity [21, 22]. In addition to assessing motivation for food, we evaluated one form of prosocial behavior (i.e., helping the experimenter pick up pens), as social exclusion has been shown to impact prosocial behaviors [23], particularly helping behavior [24] in average-weight individuals. Lastly, we set out to measure heart rate as an indicator of ANS activity and salivary cortisol as an indicator of HPA reactivity, both of which are understudied in individuals with obesity in the context of ostracism.

## Methods

### Participants

**Fig. 1:**
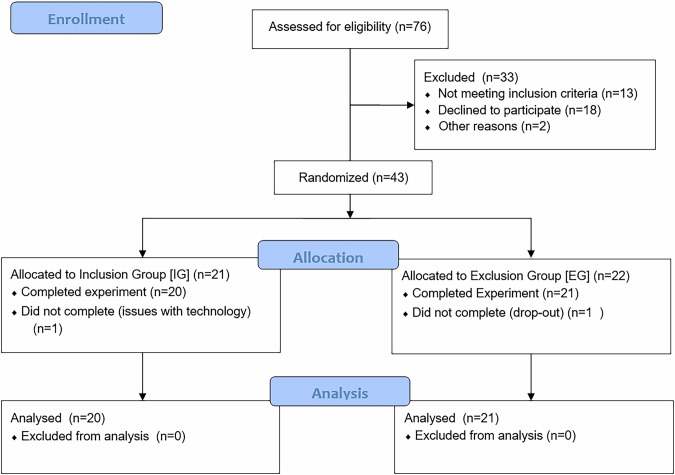
CONSORT patient flow diagram. This CONSORT patient flow depicts the selection process for the 41 children and adolescents with obesity included and randomized to either the inclusion (n=20) or exclusion (n=21) group playing the virtual Cyberball-game.

Participants were 41 children and adolescents aged 10–18 years (M_age_ = 13.37, SD_age_ = 2.46 years, 46% females) with an ICD-10-diagnosis of obesity (E66.0 [2]). Participants were recruited between summer 2021 and summer 2023 at the Outpatient Clinic for Pediatric Obesity and Dyslipidemia of the Division of Pediatric Pulmonology, Allergology and Endocrinology, Department of Pediatrics and Adolescent Medicine, see Fig. 1.

Patients were eligible if they fulfilled the following inclusion criteria: (1) 10–18 years of age, (2) an ICD-10 diagnosis of obesity due to excess calories (E66.0) with a BMI ≥97^th^ percentile [2], and (3) sufficient German language proficiency. Diagnoses were extracted a priori from the institution’s patient registry. Exclusion criteria were (1) estimated cognitive impairment (IQ < 70) and/or intellectual disability, (2) a diagnosis of schizophrenia, schizotypal or delusional disorder (F20–29), bipolar affective disorder (F31), manic episode (F30), or acute crisis (suicidality), (3) poor visual acuity, (4) a pronounced motion sickness, and (5) pregnancy or hormonal contraceptives (pill, hormonal IUD). Consistent with the inclusion and exclusion criteria, the final sample had no identified psychiatric comorbidities (also no known trauma). Additionally, there were no differences in clinical or demographic characteristics between the inclusion (*n* = 20) and the exclusion group (*n* = 21), see Table 1.

**Table 1:** Clinical and sociodemographic sample characteristics.

		Inclusion	Exclusion	Group comparison	
		[*n* = 20]	[*n* = 21]	Test statistics	*p*-value* (2-tailed)
Child characteristics					
Age, M (SD)		13.45 (2.42)	13.29 (2.55)	T = 0.211	0.834
Sex, n (%)	Female	7 (35)	12 (57)	χ^2^ = 2.020	0.155
	Male	13 (65)	9 (43)		
BMI percentile, M (SD)		98.65 (1.18)	98.29 (1.55)	T = 0.842	0.405
BMI ≥99^th^ percentile, n (%)		18 (90)	15 (71)		
Current education, n (%)	Elementary school	1 (5)	2 (10)	χ^2^ = 5.966	0.427
	Secondary school	8 (40)	5 (24)		
	Vocational secondary	7 (35)	6 (33)		
	Grammar school	4 (20)	7 (33)		
Symptom severity (SDQ), M (SD)	Overall	14.35 (3.80)	12.38 (6.32)	T = 1.215	0.233
	Emotional problems	3.45 (2.11)	3.00 (2.61)	T = 0.605	0.549
	Conduct problems	2.85 (1.60)	2.19 (1.54)	T = 1.347	0.186
	Hyperactivity	4.35 (1.53)	4.05 (2.42)	T = 0.481	0.634
	Peer problems	3.70 (1.42)	3.14 (1.71)	T = 1.132	0.265
	Prosocial behaviors	7.70 (1.84)	7.90 (2.49)	T = −0.298	0.767
Chronic stress (PSS), M (SD)	Overall	19.05 (5.10)	17.86 (6.28)	T = 0.666	0.510
	Helplessness	10.50 (4.79)	8.57 (5.37)	T = 1.212	0.233
	Self-efficacy	7.45 (2.98)	6.71 (3.51)	T = 0.722	0.475
Emotional eating (EES-C), M (SD)	Anxiety, anger	11.20 (10.44)	8.76 (9.27)	T = 0.792	0.433
	Depressive mood	10.85 (7.61)	7.33 (5.71)	T = 1.679	0.101
	Feeling unsettled	3.30 (3.05)	2.95 (2.85)	T = 0.377	0.708
Parents’ characteristics					
Marital status, n (%)	Single	2 (13)	1 (7)	χ^2^ = 0.919	0.821
	Married	9 (60)	10 (67)		
	Divorced	3 (20)	2 (13)		
	Living with new partner	1 (7)	2 (13)		
Education, n (%)	None or primary	2 (11)	4 (21)	χ^2^ = 8.598	0.377
	Vocational	9 (50)	6 (32)		
	Secondary	4 (22)	6 (31)		
	Higher	3 (17)	4 (16)		
Income not/hardly enough, n (%)		5 (30)	4 (25)	χ^2^ = 0.780	0.941

*M* mean, *SD* standard deviation, *BMI* body mass index (kg/m²), *SDQ* [30] Strengths and Difficulties Questionnaire including an overall total difficulties sum score and five subscales, *PSS* [32] Perceived Stress Scale with an overall chronic stress score and two subscales, *EES-C* [31] Emotional Eating Scale—adapted for Children and Adolescents including three subscales, *Inclusion group* participants received 33% of a total of 180 ball tosses in the Virtual Reality (VR)-Cyberball game, *Exclusion group* participants were excluded after a total of 60 tosses in the VR-Cyberball game.

*Significance at α = 0.05.

### Procedure

This study adopted a single blinded randomized experimental between-subject design (following block randomization) and was approved by the local ethics committee (vote no. 2170/2019). Potential participants were approached by study staff during their outpatient appointment. In case of approval, a separate appointment was scheduled.

Experiments were conducted in a temperature-controlled environment (22 °C) between 11:30–15:30 to account for diurnal cortisol variations. Patients were asked to abstain from eating 1 h prior to their visit, and not to consume caffeine (e.g., energy drinks) or nicotine. All patients complied with these requirements. Upon arrival, a legal guardian (a parent in all cases) provided written informed consent. Additionally, written consents (patients ≥14 years of age) or assents ( < 14 years of age) were obtained from children and adolescents. A stratified allocation scheme (by age and sex) was used by unblinded study staff to randomly assign participants to either the exclusion group or the inclusion/control group. Participants were blinded to their allocation and the study’s objectives.

Five minutes prior to the stressor, heart rate (HR) measurements (baseline) were started. Then, the head mounted display (HMD, model: HTC vive, Taiwan) was donned and participants engaged in the VR-Cyberball-game (see next section for details). Post exposure, patients recovered for 5 min (HR recovery period), were exposed to two behavioral tasks (assessing motivation for food and prosocial behavior) and proceeded to complete the last questionnaires, before they were debriefed. Patients did not receive any remuneration.

### VR-Cyberball paradigm

**Fig. 2:**
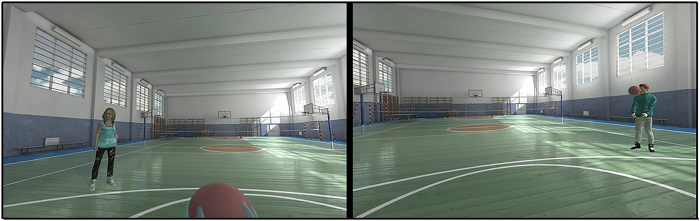
First person view of the virtual reality version of the Cyberball-paradigm. This image illustrates the participant’s first-person view of the virtual hands holding the ball and the virtual environment, with the two players positioned to the left (female) and right (male) of the participant. The setup was standardized across all participants.

To manipulate ostracism, we used the Cyberball paradigm [25], which constitutes a ball-tossing game, typically played on a desktop PC. Participants toss the ball to two animated figures until they are unexpectedly excluded from the game. This brief exclusion task is known to elicit strong emotional and physiological responses [26, 27] but has also been criticized [22, 28] for its artificial nature. By reverting to VR, which better simulates real social interactions [17, 29], we aimed to enhance ecological validity. Our VR-Cyberball game, developed in Unreal Engine 4 and displayed via a HTC Vive-headset, features two virtual agents (one male, one female) in a gymnasium (Fig. 2). Participants experience the game from a first-person view, standing upright. They may look around but not leave their spot. Controllers vibrate to indicate when it’s their turn to toss the ball. The virtual agents do not engage in any verbal or non-verbal communication. The game involves 180 tosses, with participants being excluded after 60 tosses in the exclusion condition or receiving 33% of passes (50:50 from both co-players) in the inclusion condition. The duration is 5 min for both conditions.

### Measures

#### Baseline measures

Alongside clinical data (diagnosis, height, weight), a sociodemographic questionnaire assessed age, sex, type of school, parents’ level of education, socioeconomic status, and parents’ marital status. Furthermore, symptom severity (e.g., emotional symptoms, conduct problems) was assessed with the 25-item proxy report *Strengths and Difficulties Questionnaire* (*SDQ* [30]; sdqinfo.org). The 26-item *Emotional Eating Scale – adapted for Children and Adolescents* (*EES-C* [31]) reflected the self-reported urge to eat in response to a range of negative emotions. Finally, children reported their chronic stress levels on the 10-item *Perceived Stress Scale* (*PSS* [32]).

#### Experimental measures

##### Stress

A single item on a visual analog scale (VAS, 100 mm, 0 = not at all – 100 = very much) assessed stress (“How stressed do you feel now?”) at 4 time points: (1) upon arrival (baseline), (2) during the waiting phase (anticipation), (3) after the VR-Cyberball game (stressor), (4) during the recovery phase (habituation).

##### Hunger

One item (“How strong is your urge to eat something now?”) assessed hunger (VAS, 100 mm, 0 = very small − 100 = very large; see [33]) at 4 time points: (1) baseline, (2) anticipation, (3) stressor, (4) habituation.

##### Manipulation check (MC)

The 20-item *Fundamental Social Needs Scale, FSNS* [8, 34] assessed (1) belonging, (2) self-esteem, (3) meaningful existence, and (4) control on a 5-point Likert scale (1 = not at all – 5 = extremely). Also, the two items “I was ignored”, “I was excluded” (1 = not at all – 5 = extremely) served as a MC, alongside a third item asking participants to estimate the percentage of in-game passes they received. Internal consistency (Cronbach’s α) of the sub-scales was satisfactory, ranging from Cronbach’s α = 0.70 for the sub-scale control to α = 0.85 for the sub-scale belonging. Furthermore, the 6-item *Children’s Rejection Sensitivity Questionnaire* (*CRSQ* [35, 36]) on a 6-point Likert scale (1 = not at all – 6 = very) served as a baseline measure to check for pre-existing differences between the two groups regarding their rejection sensitivity. The sum score reflects children’s expectancy to be socially rejected by others (rejection expectancy, Cronbach’s α = 0.70 in this study).

#### Physiological measures

##### Salivary cortisol

Cortisol levels [37] were quantified from commercial cotton swabs (Salivette®, Sarstedt, Nuembrecht, Germany) using Elecsys Cortisol II electrochemiluminescence (ECLIA) assays (Roche, Rotkreuz, Switzerland) on cobas®e801 analyzers (Roche). The measuring interval of this test ranges from 0.054 µg/dL–634 µg/dL (conversion to nmol/L = µg/dLx27.586) and the manufacturer reports inter-assay precision in human saliva to be between 1.3–4.9% CV (coefficient of variation) and intermediate precision to be between 1.9–7.8% CV. Participants put the swab into their cheek pouch for 120 s at a resting-state-phase and post-exposure to VR-Cyberball. Analyses were conducted at the certified (ISO 9001) and accredited (ISO 15189) Department of Laboratory Medicine, Medical University of Vienna.

##### Heart rate (HR)

A chest belt with a wireless sensor (Polar V800, Polar Electro, Finland) measured HR for 15 min as an indicator of ANS activity (5 min baseline, 5 min VR-Cyberball, 5 min habituation). Data were pre-processed via visual inspection of artefacts. Subsequently, mean HR and heart rate variability (HRV, root mean square of successive difference, rMSSD) were calculated based on the Task Force of the European Society of Cardiology and the North American Society of Pacing and Electrophysiology recommendations [38]. Patients were standing up during baseline and stressor but sat down for the habituation phase.

#### Behavioral measures

##### Self-regulatory behaviors

Similar to past experiments [5, 10, 11], we used the reinforcing value of unhealthy snacks as an indicator of self-regulation. Like in Baumeister et al. [10], a bowl containing a large serving (1728 g) of bite-size high-calorie sweets (e.g., Twix, Maoam, PEZ*;* see supplement) was placed in front of participants post VR-Cyberball. Yet, contrary to past research [5, 10, 11], participants did not actually consume (or taste-test [10, 11]) these high-calorie foods. After consulting with the medical obesity team, we concluded that this approach was inappropriate due to our participants’ health conditions and the potential to interfere with their ongoing medical care. Participants were instructed to take as many or as few sweets as they wished to consume at this point in time and place them in a plastic bag (instead of eating them on the spot). The bag was then collected by study-staff and weighed. The serving’s weight (in grams) served as an indicator of motivation for high-calorie food consumption, with a higher weight reflecting greater motivation for sweets and, consequently, poorer self-regulation. At the end of the experiment, participants were debriefed about this study’s measures.

##### Helping behaviors

To assess one form of prosocial behavior post-exclusion, the experimenter accidentally dropped a cup of pens in the participant’s imminent vicinity [21]. Helping the experimenter to pick up the pens (y/n) and the time to pick them up, both served as indicators of prosocial behavior.

### Statistical methods

Analyses were conducted using IBM SPSS 29, and R 4.4.2. Linear mixed-effects models were used to analyze primary outcomes – including salivary cortisol, heart rate (HR), heart rate variability (HRV), stress, and urge to eat – while accounting for repeated measures nested within participants. These models included fixed effects for time, group, and their interaction, with random intercepts to capture individual variability. T-tests (or Welch’s t-tests when the assumption of homogeneity of variances was violated), as well as χ²-tests, were used to analyze the secondary outcomes of fundamental social needs, self-regulatory behavior, and helping behavior. Tukey-corrected simple effects analyses were applied whenever interactions reached significance. The sample size needed was established a priori. Power analysis using G*Power [39] with α = 0.05 and 1-β = 0.80 indicated that our study was sufficiently powered; that is, we expected small to moderate effect sizes (Cohen’s d = 0.40) for group comparisons and the time*group interaction, which requires an N of 28–42 to be considered clinically significant. In our study context, a Cohen’s d of 0.4 corresponds to a difference of approximately 5 beats per minute (bpm) in HR or about 9 rating points on the urge-to-eat scale between the two conditions at a specific time point. While these differences may appear modest, a 5 bpm change in HR can be physiologically significant and a 9-point difference on a 0–100 scale likely indicates a meaningful behavioral shift, providing a practical benchmark for clinical relevance.

## Results

### Manipulation check

**Fig. 3:**
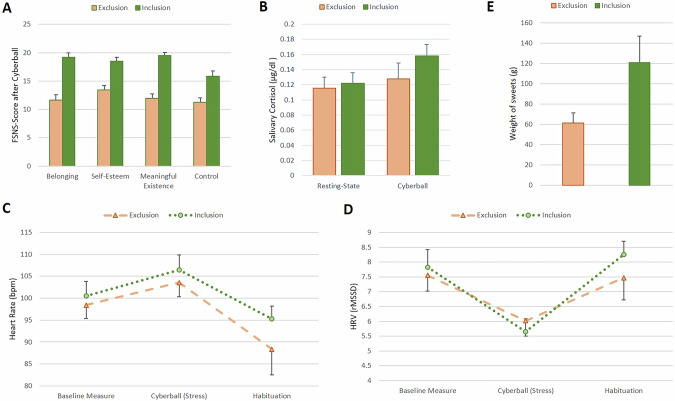
Group differences in primary and secondary outcomes. **A** FSNS Fundamental Social Needs Scale [8, 34] with four sub-scales; **B** Salivary cortisol in micrograms (µg) per deciliter (dl). **C** Heart rate in beats per minute (bmp); y-axis capped at 80 bpm for brevity **D** HRV heart rate variability, rMSSD root mean square of successive difference, y-axis capped at 4 rMSSD for brevity; **E** Weight of sweets in gram (g).

There were no differences in clinical and demographic characteristics between conditions (Table 1). Furthermore, there was no significant difference regarding self-reported rejection expectancy (CRSQ) between the groups (see Table 2 for the statistical results for each outcome). Included participants reported receiving significantly more ball tosses than excluded participants (32% vs. 13%). Moreover, as shown in Fig. 3A, excluded participants reported significantly lower satisfaction of fundamental social needs – specifically, lower levels of belonging, self-esteem, meaningful existence, and control – compared to those in the inclusion condition.

**Table 2:** Summary of statistical analyses.

Outcome	Comparison/Effect	Test statistic (t, χ²)	*p*-value*
Manipulation check			
Rejection expectancy	Group difference	t_39_ = 1.702	0.097
Estimated ball tosses	Group difference	t_28.178_ = 5.315	<0.001
Fundamental social needs			
Belonging	Group difference	t_39_ = 5.906	<0.001
Self-esteem	Group difference	t_39_ = 4.488	<0.001
Meaningful existence	Group difference	t_39_ = 7.974	<0.001
Control	Group difference	t_39_ = 3.800	<0.001
Self-reported urge to eat	Time	χ²_3_ = 1.136	0.768
	Group	χ²_1_ = 0.302	0.582
	Time × Group	χ²_3_ = 2.097	0.553
Self-reported stress	Time	χ²_3_ = 0.796	0.850
	Group	χ²_1_ = 1.947	0.163
	Time × Group	χ²_3_ = 0.222	0.947
Salivary cortisol	Time	χ²_1_ = 19.010	<0.001
	Group	χ²_1_ = 1.663	0.197
	Time × Group	χ²_1_ = 4.143	0.042
Heart rate	Time	χ²_2_ = 7.268	0.026
	Group	χ²_1_ = 0.148	0.701
	Time × Group	χ²_2_ = 0.814	0.666
Heart rate variability (rMSSD)	Time	χ²_2_ = 41.849	<0.001
	Group	χ²_1_ = 0.110	0.740
	Time × Group	χ²_2_ = 3.666	0.160
Self-regulatory behavior	Group difference	t_24.411_ = 2.123	0.044
Helping behavior (dichotomous)		χ²_1_ = 3.881	0.054
Helping Behavior (time until help)		t_31_ = 0.518	0.608

Comparison between inclusion group (*n* = 20) and exclusion group (*n* = 21); *rejection expectancy* measured with baseline CRSQ [35, 36], *estimated ball tosses* a single item asking participants to estimate the percentage of received in-game passes, *fundamental social needs* measured with FSNS [8, 34], *self-reported urge to eat* single VAS-item, *self-reported stress* single VAS-item, *rMSSD* root mean square of successive difference, *Self-regulatory behavior* weight of sweets picked post-VR-Cyberball, *Helping behavior* helping experimenter pick up pens: yes or no (dichotomous), and time until participants started to pick up pens (in seconds).

*Significance at α = 0.05.

### Self-reported urge to eat and stress

Additionally, we analyzed the urge to eat and stress across four time points. For urge to eat, linear mixed-effects models showed no significant main effect of time, group, as well as no time*group interaction. Similarly, for self-reported stress, we found no significant effect of time, no between-group effect, and no time*group interaction. See Table 2 for statistical results.

### Physiological reactivity

Results indicate a significant increase in salivary cortisol after social exclusion compared to the resting-state phase. Additionally, a significant time*group interaction effect demonstrated that the excluded group exhibited significantly lower salivary cortisol reactivity compared to the included group. No significant between-group effect for salivary cortisol was observed. Additionally, a significant increase in HR was observed during the task across groups; however, no significant time*group interaction was found for HR, and no between-group differences were detected. Similar results were found for HRV represented by rMSSD values, with a decrease in HRV during the task in all participants, neither significant between-group effects nor a significant time*group interaction were observed; see Fig. 3B–D and Table 2.

### Behavioral measures

A significant group difference was observed regarding self-regulatory behavior after VR-Cyberball. As illustrated in Fig. 3E, excluded participants picked significantly fewer grams of sweets than included participants. Helping behavior, as measured by the pen task, tended to be higher in excluded compared to included participants, though this difference did not reach statistical significance (*p* = 0.054). This effect was limited to the decision to help rather than the latency to help.

## Discussion

Consistent with prior studies [5, 8, 17, 21], being ostracized in VR led to a significant depletion of fundamental social needs like belonging, self-esteem, meaningful existence, and control in children and adolescents with obesity (BMI ≥97^th^ [2]). However, we did not find that experiencing social exclusion deteriorated self-regulatory eating behaviors [5, 9, 14]. In our study, excluded participants showed less motivation for high-calorie foods than those in the inclusion/control condition.

While it is commonly assumed that ostracism draws attentional resources away from self-monitoring and impairs one’s control over food intake [5], our study suggests the opposite – at least for an experimental manipulation which did not entail actually eating high-calorie foods [5, 10, 11]. Due to the health condition of our participants (BMI ≥97^th^ percentile [2]), including those with more severe obesity (BMI ≥99^th^ percentile), we decided to measure motivation for food, rather than actual intake. Unlike previous studies [10, 11] which masked food intake by framing it as a taste-test to obtain an incidental measure of self-regulatory behaviors, we offered our participants a large serving of sweets and allowed them to take as many or as few as they wished to consume at a given time. Hence, our participants were aware of collecting unhealthy foods, and this awareness may explain the differences in results between our study and previous research. In circumstances in which the self, or parts of the self, contribute to the exclusion, an adaptive response may entail altering this part of the self to regain social acceptance. Potential responses to ostracism may thus include increased conformity [34], such as reducing food intake or engaging in more prosocial behavior [40]. In addition to the lesser servings of sweets, we also observed that excluded participants tended to help the experimenter more frequently. Although this was only a trend, together with the smaller servings, it may be indicative of participants’ adaptive response to ostracism. Future research should establish whether a heightened attentional focus on restoring social acceptance could help explain differences in self-regulation.

This is the first study to consider both HPA and ANS reactivity to ostracism in pediatric patients with BMI ≥97^th^ percentile. While HR levels were increased during the Cyberball-game but showed no differences between the inclusion and exclusion condition, salivary cortisol levels were significantly higher in included individuals than in excluded ones. The fact that HPA axis and ANS reactivity remain understudied in the context of obesity [3, 41], particularly in children, complicates the contextualization of our results. Similar to our findings, adult studies show increased HR during social exclusion [17, 42] and blunted cortisol responses in ostracized women [43, 44]. Furthermore, research suggests that the presence of trauma (i.e., adverse childhood events, post traumatic stress disorder, PTSD) may be associated with a blunted cortisol response towards social stress, leading to a diminished capacity of the body to regain homeostasis and hence, to prolonged periods of stress [45, 46]. In the context of our study, this may help further explain the lowered motivation for food in excluded individuals. However, similar to trauma, excess fat tissue has also been linked to chronically downregulated HPA and ANS in overweight children, causing lower overall ANS activity and blunted cortisol responses compared to average-weight peers [3, 41]. These complex interactions highlight the need for more social stress research in children and adolescents with obesity, all the more given the pivotal role of HPA in the disease’s etiology [47].

### Limitations

Our results must be interpreted in relation to several limitations. First, we classified participants based on the ICD-10 E-Diagnoses (e.g., E66.0 [1, 2]), commonly used in Europe, particularly in Germany and Austria. However, as of 2024, the U.S. has adopted the ICD-10-CM Z-codes [48, 49], which provide a more detailed classification. For instance, class 3 obesity (Z68.56) is defined as a BMI ≥ 140% of the 95th percentile [48, 49]. Comparisons between studies using these different classification systems should therefore be interpreted with caution. Furthermore, our sample was small but ensured adequate statistical power. However, replicating the study in larger samples, particularly with a more balanced distribution of sex, as well as focusing exclusively on children and adolescents with severe obesity, who may face different challenges to those with less severe obesity [15, 16], would be beneficial. Second, the experimental design may to some degree limit the ecological validity of our results. In everyday life there are more alternatives to coping with social stress than just food [5]. The context in which stress is experienced, as well as available social resources like significant others may also have an impact on the ability to self-regulate under stress [50]. Collecting data in real-life circumstances with Ecological Momentary Assessment [51] may better be suited to reflect the complex association between social stress and depleted self-regulatory behaviors regarding high-calorie food consumption. Finally, we measured acute, short-term physiological stress responses. Yet, as chronically elevated cortisol levels may increase appetite, insulin secretion, and fat accumulation in the long-term [3], collecting scalp hair samples as indicators of systemic cortisol secretion [52] may be an additional route of action for this field of research.

## Conclusion

In conclusion, our study is the first to examine ostracism in relation to salivary cortisol and heart rate in patients with obesity (BMI ≥97^th^ percentile). Using self-report, physiological and behavioral measures to cover a broad range of responses, we found that the painful experience of being ostracized led to a diminished motivation for food in children and adolescents with excess weight, a possible motive being the need to replenish social resources. Based on these results, we may hypothesize that affiliative behaviors might play a role in dietary decisions, especially in the face of social stress. Clinical interventions could thus incorporate social support strategies, such as peer groups, to help mitigate the emotional impact of social exclusion experiences on dietary behaviors. Additionally, psychological therapies like cognitive behavioral therapy (CBT), can foster resilience against social stress and promote healthier eating patterns. Furthermore, future research should take a broader social context into account, also probing intimate relationships with family and friends, to learn more about the dynamics between depleted fundamental needs, physiological reactivity, and self-regulatory behaviors in the hope of contributing to the notoriously difficult treatment of pediatric patients with obesity.

## Supplementary information

Table A. Experimental sweets used in the experiment.

## Data Availability

Research data are not in the public domain as they contain sensitive patient data. Data may be requested by contacting the corresponding author.
