# Supplementary material for: Ostracism, cortisol reactivity, and motivation for high-calorie food in children and adolescents with obesity
Source: Int J Obes (Lond). 2025 Sep 24;49(10):2117–24. doi: 10.1038/s41366-025-01824-3 (PMC12532573; doi:10.1038/s41366-025-01824-3)
Supplement: Supplementary file 1 — Table A. Experimental sweets used in the experiment. [file 41366_2025_1824_MOESM1_ESM.docx]

| **Type of sweets** | **Serving  (g)** | **Kilocalories  (kCal)** | **Fat  (g)** | **Carbo-hydrates (g)** | **Protein  (g)** |
| --- | --- | --- | --- | --- | --- |
| Bounty | 11 | 54 | 2.9 | 6.5 | 0.4 |
| Nimm2 | 6 | 22 | 0.0 | 5.5 | 0.0 |
| Fizzers | 11 | 43 | 0.3 | 9.6 | 0.0 |
| Twix | 11 | 54 | 2.6 | 7.2 | 0.5 |
| Maoam | 5 | 20 | 0.3 | 4.2 | 0.1 |
| Galaxy | 9 | 43 | 2.2 | 5.5 | 0.5 |
| Schokobon | 7 | 33 | 2.1 | 3.0 | 0.5 |
| Schokobon white | 7 | 33 | 2.2 | 2.7 | 0.6 |
| Mars | 10 | 44 | 1.6 | 7.1 | 0.4 |
| Snickers | 11 | 54 | 2.5 | 6.8 | 0.9 |
| Teasers | 9 | 54 | 3.1 | 5.8 | 0.7 |
| PEZ | 8 | 32 | 0.1 | 7.6 | 0.0 |

**Appendix**

**Table A. Experimental sweets used in the experiment.**

*Note:* The nutritional information is to be understood per serving, see first column. The weight of the overall serving used in this study for all sweets was 1728g.
